# Supplementary material for: Assessing changes in mood state in university students following short-term study abroad
Source: PLoS One. 2021 Dec 23;16(12):e0261762. doi: 10.1371/journal.pone.0261762 (PMC8699600; doi:10.1371/journal.pone.0261762)

PLOS ONE

Assessing changes in mood state in university students following short-term study abroad

--Manuscript Draft--

**Manuscript Number:**	PONE-D-21-12484
**Article Type:**	Research Article
**Full Title:**	Assessing changes in mood state in university students following short-term study abroad
**Short Title:**	Assessing changes in mood state in university students following short-term study abroad
**Corresponding Author:**	Tsukasa Yamanaka, Ph.D.<br>Ritsumeikan University - Biwako Kusatsu Campus: Ritsumeikan Daigaku - Biwako Kusatsu Campus<br>Kusatsu, Shiga JAPAN
**Keywords:**	Profile of Mood States (POMS); Satisfaction With Life Scale (SWLS); Personality traits (NEO-FFI); Short-term study-abroad; Mood state
**Abstract:**	Short-term study-abroad (STSA) programs provide an alternative for students who would otherwise not engage in international academic activities. Though improvements in the levels of intercultural sensitivity and general academic aspects attained by STSA programs have been examined, little is known about the impact they have on the mood state of students. Here, we examined changes in mood state associated with participation in a cohort of Japanese university students. Mood state was assessed using the Profile of Mood States (POMS), the Satisfaction With Life Scale (SWLS) and the Gratitude Questionnaire (GQ-6). Results indicated that the POMS mean scores of Vigor-Activity and SWLS peaked immediately following participation in the STSA program, and remained at comparable levels for up to one month. These results suggest that STSA programs positively enhance the mood state of college students, indicating that the benefits arising from participation may extend beyond those typically reported in the academic domain.
**Order of Authors:**	Tsukasa Yamanaka, Ph.D.
	Noriko Yamagishi
	Norberto Eiji Nawa
	Stephen J Anderson
**Additional Information:**	
**Question**	**Response**
**Financial Disclosure**<br><br>Enter a financial disclosure statement that describes the sources of funding for the work included in this submission. Review the submission guidelines for detailed requirements. View published research articles from *PLOS ONE* for specific examples.<br><br>This statement is required for submission and **will appear in the published article** if the submission is accepted. Please make sure it is accurate.	Initials of the authors who received each award<br>-TY, NY<br><br>Grant numbers awarded to each author<br>-N/A<br><br>The full name of each funder<br>-Ritsumeikan Inamori Philosophy Research Center<br><br>URL of each funder website<br>-http://www.ritsumei.ac.jp/research/riprc/eng/<br><br>Did the sponsors or funders play any role in the study design, data collection and analysis, decision to publish, or preparation of the manuscript?<br>-NO.

Powered by Editorial Manager® and ProduXion Manager® from Aries Systems Corporation

**Unfunded studies**<br>Enter: *The author(s) received no specific funding for this work.*<br><br>**Funded studies**<br>Enter a statement with the following details:<br>• Initials of the authors who received each award<br>• Grant numbers awarded to each author<br>• The full name of each funder<br>• URL of each funder website<br>• Did the sponsors or funders play any role in the study design, data collection and analysis, decision to publish, or preparation of the manuscript?<br>• **NO** - Include this sentence at the end of your statement: *The funders had no role in study design, data collection and analysis, decision to publish, or preparation of the manuscript.*<br>• **YES** - Specify the role(s) played.<br><br>* typeset	
**Competing Interests**<br><br>Use the instructions below to enter a competing interest statement for this submission. On behalf of all authors, disclose any competing interests that could be perceived to bias this work—acknowledging all financial support and any other relevant financial or non-financial competing interests.<br><br>This statement is required for submission and **will appear in the published article** if the submission is accepted. Please make sure it is accurate and that any funding sources listed in your Funding Information later in the submission form are also declared in your Financial Disclosure statement.<br><br>View published research articles from *PLOS ONE* for specific examples.	NO authors have competing interests.

**NO authors have competing interests**<br><br>Enter: *The authors have declared that no competing interests exist.*<br><br>**Authors with competing interests**<br><br>Enter competing interest details beginning with this statement:<br><br>*I have read the journal's policy and the authors of this manuscript have the following competing interests: [insert competing interests here]*<br><br>* typeset	
**Ethics Statement**<br><br>Enter an ethics statement for this submission. This statement is required if the study involved:<br><br>• Human participants<br>• Human specimens or tissue<br>• Vertebrate animals or cephalopods<br>• Vertebrate embryos or tissues<br>• Field research<br><br>Write "N/A" if the submission does not require an ethics statement.<br><br>General guidance is provided below. Consult the submission guidelines for detailed instructions. **Make sure that all information entered here is included in the Methods section of the manuscript.**	Human Subject Research (involving human participants and/or tissue)<br><br>Give the name of the institutional review board or ethics committee that approved the study<br>-Ritsumeikan Inamori Philosophy Research Center, General Committee<br><br>Include the approval number and/or a statement indicating approval of this research<br>-Ritsumeikan Inamori Philosophy Research Center, General Committee's approval<br><br>Indicate the form of consent obtained (written/oral) or the reason that consent was not obtained (e.g. the data were analyzed anonymously)<br>- Oral consent was obtained from the participants.

**Format for specific study types**<br><br>**Human Subject Research (involving human participants and/or tissue)**<br>• Give the name of the institutional review board or ethics committee that approved the study<br>• Include the approval number and/or a statement indicating approval of this research<br>• Indicate the form of consent obtained (written/oral) or the reason that consent was not obtained (e.g. the data were analyzed anonymously)<br><br>**Animal Research (involving vertebrate animals, embryos or tissues)**<br>• Provide the name of the Institutional Animal Care and Use Committee (IACUC) or other relevant ethics board that reviewed the study protocol, and indicate whether they approved this research or granted a formal waiver of ethical approval<br>• Include an approval number if one was obtained<br>• If the study involved *non-human primates*, add *additional details* about animal welfare and steps taken to ameliorate suffering<br>• If anesthesia, euthanasia, or any kind of animal sacrifice is part of the study, include briefly which substances and/or methods were applied<br><br>**Field Research**<br><br>Include the following details if this study involves the collection of plant, animal, or other materials from a natural setting:<br>• Field permit number<br>• Name of the institution or relevant body that granted permission	
**Data Availability**<br><br>Authors are required to make all data underlying the findings described fully available, without restriction, and from the time of publication. PLOS allows rare exceptions to address legal and ethical concerns. See the PLOS Data Policy and FAQ for detailed information.	No - some restrictions will apply

A Data Availability Statement describing where the data can be found is required at submission. Your answers to this question constitute the Data Availability Statement and **will be published in the article**, if accepted.<br><br>**Important:** Stating 'data available on request from the author' is not sufficient. If your data are only available upon request, select 'No' for the first question and explain your exceptional situation in the text box.<br><br>Do the authors confirm that all data underlying the findings described in their manuscript are fully available without restriction?	
**Describe where the data may be found in full sentences. If you are copying our sample text, replace any instances of XXX with the appropriate details.**<br><br>• If the data are **held or will be held in a public repository**, include URLs, accession numbers or DOIs. If this information will only be available after acceptance, indicate this by ticking the box below. For example: *All XXX files are available from the XXX database (accession number(s) XXX, XXX.*).<br>• If the data are all contained **within the manuscript and/or Supporting Information files**, enter the following: *All relevant data are within the manuscript and its Supporting Information files.*<br>• If neither of these applies but you are able to provide **details of access elsewhere**, with or without limitations, please do so. For example:<br><br>*Data cannot be shared publicly because of [XXX]. Data are available from the XXX Institutional Data Access / Ethics Committee (contact via XXX) for researchers who meet the criteria for access to confidential data.*<br><br>*The data underlying the results presented in the study are available from (include the name of the third party*	Data are available from the Ritsumekan University Institutional Data Access (contact person: Prof. Tsukasa Yamanaka, yaman@fc.ritsumei.ac.jp) for researchers who meet the criteria for access to confidential data.

Powered by Editorial Manager® and ProduXion Manager® from Aries Systems Corporation

and contact information or URL).<br>• This text is appropriate if the data are owned by a third party and authors do not have permission to share the data.<br><br>* typeset	
Additional data availability information:	

Click here to access/download;Manuscript;20210415.docx

Assessing changes in mood state in university students following short-term study abroad

Tsukasa Yamanaka[13¶*]

Noriko Yamagishi[234¶]

Norberto Eiji Nawa[35¶]

Stephen J. Anderson[6¶]

[1] College of Life Sciences, Ritsumeikan University, Kusatsu, Shiga, Japan

[2] College of Global Liberal Arts, Ritsumeikan University, Ibaraki, Osaka Japan

[3] National Institute of Information and Communications Technology, Suita, Osaka, Japan

[4] Ritsumeikan Inamori Philosophy Research Center, Ibaraki, Osaka, Japan

[5] Graduate School of Frontiers Biosciences, Osaka University, Suita, Osaka, Japan

[6] School of Life and Health Sciences, Aston University, Birmingham, UK

[*]Corresponding author:

E-mail: yaman@fc.ritsumei.ac.jp (TY)

[¶]These authors contributed equally to this work.

Abstract

Short-term study-abroad (STSA) programs provide an alternative for students who would otherwise not engage in international academic activities. Though improvements in the levels of intercultural sensitivity and general academic aspects attained by STSA programs have been examined, little is known about the impact they have on the mood state of students. Here, we examined changes in mood state associated with participation in a cohort of Japanese university students. Mood state was assessed using the Profile of Mood States (POMS), the Satisfaction With Life Scale (SWLS) and the Gratitude Questionnaire (GQ-6). Results indicated that the POMS mean scores of Vigor-Activity and SWLS peaked immediately following participation in the STSA program, and remained at comparable levels for up to one month. These results suggest that STSA programs positively enhance the mood state of college students, indicating that the benefits arising from participation may extend beyond those typically reported in the academic domain.

Introduction

Though the multifaceted benefits of participation in study-abroad programs are well documented [1], the percentage of Japanese students engaging in international academic programs remains low at approximately 1% [2]**.** To counter that low take-up, universities in Japan have developed a variety of study-abroad programs [3] to increase the number of domestic students gaining international academic experience by targeting subgroups of the student population that are less likely, or more reluctant, to seek international experience during their college years. Such efforts are believed to help foster more “globalized” academic environments, which are thought to be an important prerequisite for prospective domestic and international students [4-5]. To help students overcome the barriers that may prevent their participation in STSA programs, universities have resorted to different strategies, such as minimizing language requirements or offering access to financial support.

It is generally reported that long-term study-abroad programs, lasting one or more semesters, bring greater benefits than short-term (STSA) programs [6]. However, [3] argued that STSA programs should not be overlooked as they target a subgroup of the student

population that would otherwise not engage in any type of international academic activity. Moreover, STSA programs provide a more accessible and affordable alternative than long-term programs. [7] have noted that the majority of more recent study abroad-programs have a short-term character. As a result of this trend, most universities in Japan now offer STSA programs as part of their official curricula.

The benefits associated with study-abroad programs have been measured in various ways, including both intercultural sensitivity [5] and desire to attend graduate school [6], which are fundamental issues in the context of international education. Assessing the long-term impact of international study, Yokota et al. [8] reported that students who undertook study abroad, compared with those who did not, adapted better to new and different cultural environments. Notably, this positive outcome was associated not only with advanced language or technical skills but also with fundamental competences of emotional intelligence and socialization, leaving students with an overall progressive outlook on life.

Other studies have shown positive effects in how participants of STSA programs perceive themselves in a globalized context after obtaining international experience. Using large-scale surveys, [9] reported that students who took part in a five-week STSA exchange program had higher levels of self-assessed "global awareness" when compared with students who only took classes about intercultural understanding in their country of origin. [10] examined participants undertaking a three- to four-week STSA program, and assessed notions associated with global consciousness before, during and after the program. Their results indicated that a participant's self-assessed acquired practical knowledge about the world was significantly enhanced on completion of the program.

Few studies have examined the value of more time-limited exchange programs, such as those lasting only one week [6, 11], and how such programs compare with those of longer duration. However, given the poor take-up of international study by Japanese students [12], and the increasing costs of study-abroad programs, a full investigation of the effects of time-limited STSA programs appears warranted.

Our goal in this paper was to examine changes in the mood state of individuals participating in a one-week STSA program. We employed metrics that are typically applied in experimental psychology (see Methods for details), but which have thus far been less favored

in the field of study-abroad research [13-14]. To examine the impact on the mood state of students participating in the one-week STSA program, data collection was performed at two time-points before and two time-points after their time abroad.

Methods

Participants

The target sample group consisted of 40 (24 males, 16 females) Japanese undergraduate students attending a Japanese university. They participated in a short-term study-abroad program of one week duration, called the Global Fieldwork Project (more details in section below). Students taking part in the program were enrolled in several different majors: Letters (30%); Economics (17.5%); Sociology (15%); Life Sciences (7.5%); Policy Science (7.5%); Gastronomy Management (5%); Business Administration (5%); Science and Engineering (5%); Comprehensive Psychology (2.5%); International Relations (2.5%); and Information Science and Engineering (2.5%). A total of 42.5% of participants in the STSA program were freshmen, 40% were sophomores, 15% were juniors and 2.5% were seniors. The program had two destinations, with 65% of the students conducting the fieldwork in Penang, Malaysia, and 35% conducting the fieldwork in Phnom Pen, Cambodia.

NEO-Five Factor Inventory (NEO-FFI)

To ensure that participants of the STSA program were a representative student sample, we asked participants to rate items in the NEO Five-Factor Inventory (NEO-FFI, [15]), and compared them with the scores given by a group of students from the same university who neither participated in the program nor spent any time overseas during the same period. There were 39 undergraduate students (17 males, 22 females) in the comparison group, comprised of 35.9% from the College of Life Sciences and 46.1% from the College of Pharmaceutical Sciences (25.6% freshmen, 74.4% sophomores). The NEO-FFI is a shortened version of the NEO Personality Inventory-Revised, an implementation of an empirically validated five-factor model of human personality [16]. The FFI is one of the most extensively applied models of personality currently in use [17], describing individual differences in terms of five personality

traits: Neuroticism, Extraversion, Openness, Agreeableness, and Conscientiousness. This personality model has been examined using both cross-sectional and longitudinal studies on populations of different ages and cultural backgrounds [18-20].

Profile of Mood States (POMS)

The mood states of participants were assessed using a Japanese translation of the Profile of Mood States (Second Edition, Adult Short Form, POMS 2®, Kanekoshobo Inc., Tokyo, Japan). The POMS was designed to assess the mood states of individuals aged 13 years and older [21]. It consists of a collection of self-rating scales to score various aspects related to mood and feelings experienced, allowing for a quick assessment of transient, fluctuating feelings, as well as enduring affective states. The POMS is composed of six scales: Anger-Hostility (AH), Confusion-Bewilderment (CB), Depression-Dejection (DD), Fatigue-Inertia (FI), Tension-Anxiety (TA), and Vigor-Activity (VA). Higher POMS scores indicate greater intensity of the corresponding composite construct. Note that only VA has a positive valence, while all other scales have a negative connotation. An aggregate score of total mood disturbance (TMD) can be computed based on the six scores, with greater scores indicating higher disturbance. The POMS individual scales, or the TMD score, have been employed to monitor natural changes in mood state or alterations in mood state following behavioral interventions in clinical, athletic and psychology research settings [22-24]. A few studies have employed the POMS in researching study-abroad programs (e.g. [25]).

Satisfaction With Life Scale (SWLS)

The Satisfaction With Life Scale (SWLS; [26]) consists of five items developed to measures an individual’s satisfaction with life as a whole, regardless and beyond specific domains such as health and finances. Satisfaction with life is thought to be a fundamental component of the construct of subjective well-being [27]. The SWLS has been largely applied to clinical and non-clinical populations across different cultural contexts [28-29].

Gratitude Questionnaire (GQ-6)

The Gratitude Questionnaire (GQ-6) is a six-item scale used to assess the individual disposition of experiencing the emotion of gratitude, conceptualized as an affective trait reflecting one's tendency to attend and respond to the role of other people in giving rise to positive outcomes that benefit the self [30]. GQ-6 scores have been found to be positively correlated with SWLS scores [31], as well as subjective scores of happiness [32] and job satisfaction [33].

Global Fieldwork Project (GFP)

The GFP is a project-oriented, fieldwork-based program, structured such that students first explore possible themes of interest and concerns regarding the country they are about to visit, and then pursue those themes with the assistance of local university students ("buddies") during the STSA period. The program began with a classroom orientation session at the end of June (i.e., about the time when the spring term ends and the summer vacation begins).

In the initial orientation session of one day duration, students were required to form groups of three, discuss possible topics of study, and devise a fieldwork plan to execute during the STSA. The topics were quite diverse, reflecting their interests and backgrounds. Students were encouraged to form hypotheses about various socioeconomic aspects of the country they were about to visit.

Approximately one month after the orientation session, students traveled to Malaysia or Cambodia – the two destinations of the STSA – led by a faculty member whose role was to oversee the entire program and provide assistance to the students when necessary.

During the study abroad period, their work consisted of gathering first-hand information to test their hypotheses, achieved through questionnaires and interviews with local people. The interviews were conducted in collaboration with their assigned "buddies". Apart from the group-based fieldwork, the program included an introductory lecture by a local university professor, an orientation session and a farewell party. Excluding travel time to and from the overseas destination, the total period spent abroad was six days.

Two to four weeks after their return from overseas, students were required to present their findings in front of an invited audience of fellow students and academics. As part of their presentation, they reported on whether or not their initial hypotheses about the socioeconomic

conditions of the visited country were confirmed. Through personal interviews, students also reported about their overall experience of the GFP and the specific study period.

Study Design

During the orientation day, approximately one month prior to the scheduled departure date (Time 1, T1), participants were requested to answer the items in the POMS, SWLS, GQ-6 and NEO-FFI. In order to mask the long-term goal of these tasks, participants were instructed to answer the items as part of their normal classroom activities. The same was true for the comparison group when completing the NEO-FFI.

Because the current study spanned no more than three months, the NEO-FFI was completed only once. The POMS, SWLS and GQ-6 were completed four times in total: (i) during the orientation day; (ii) one day before departure (T2, mid-August for the students heading to Cambodia; early September for the students heading to Malaysia); (iii) the day they arrived back in Japan (T3, end of August for students that visited Cambodia; mid-September for students that visited Malaysia); and (iv) during the final presentation session, held over a single weekend at the end of September (T4).

Statistical analysis

We employed a two-way, repeated measures analysis of variance (rm-ANOVA) with group (STSA and comparison groups) as a between-subjects factor, and the NEO-FFI traits (Neuroticism, Extraversion, Openness, Agreeableness, and Conscientiousness) as within-subject factors. We employed a one-way rm-ANOVA to examine the individual components of the POMS mood state (Anger-Hostility, Confusion-Bewilderment, Depression-Dejection, Fatigue-Inertia, Tension-Anxiety, and Vigor-Activity), with time (T1, T2, T3, T4) as a within-subject factor. A similar analysis was performed using the data from the SWLS and GQ6. Degrees of freedom were adjusted using Greenhouse-Geisser estimates of sphericity (Mauchly's sphericity test). All analyses were performed using SPSS version 25 (IBM, New York, USA).

Results

NEO-FFI trait differences between study-abroad and comparison groups

We first examined whether our study-abroad group differed from the comparison group on the five NEO-FFI traits. A two-way repeated measures ANOVA revealed a significant main effect for NEO-FFI traits ($F(2.289, 173.974) = 4.327$, $p = 0.011$, Greenhouse-Geisser corrected), but no main effect of group ($F(1, 76) = 2.442$, $p = 0.122$). Moreover, there was no interaction between the NEO-FFI traits and group ($F(2.289, 173.974) = 0.2401$), $p = 0.816$). Post-hoc tests using Bonferroni correction revealed that the mean score for the NEO-FFI Extraversion trait ($M = 25.78$, $SD = 8.63$) and Agreeableness trait ($M = 30.24$, $SD = 5.87$) differed significantly ($p < 0.001$). These results provide confirmatory evidence that there were no latent personality trait differences between the study-abroad and comparison groups.

POMS

Vigor-Activity (VA) scores across time

We entered the scores of the component VA in a one-way rm-ANOVA, with time (T1, T2, T3, T4) as a within-subjects factor. The results showed a significant main effect of time ($F(3, 117) = 24.078$, $p < 0.0001$). Post-hoc tests using Bonferroni correction for multiple comparisons indicated that the mean VA score at time T3 ($M = 13.67$, $SD = 4.621$), collected the day after participants returned from abroad, was larger than the mean score at both time T1 ($M = 9.08$, $SD = 4.891$), T2 ($M = 8.98$, $SD = 4.452$) and T4 ($M = 11.53$, $SD = 3.883$). Furthermore, the mean score at T4 was larger than the scores collected at T1 and T2, and smaller than the score at T3. These results are shown in Fig 1 (a).

Fig 1. POMS scores across time. (a) Vigor-Activity (VA), (b) Confusion-Bewilderment (CB), (c) Fatigue-Inertia (FI), and (d) Tension-Anxiety (TA). T1: one month before departure overseas, T2: one day before departure, T3: the day of arrival back in Japan, and T4: 2-4 weeks after their return. The asterisk (*) indicates statistically significant

pairwise differences ($p < 0.05$ level), corrected for multiple comparisons using the Bonferroni method.

Confusion-Bewilderment (CB), Fatigue-Inertia (FI) and Tension-Anxiety (TA) scores across time

Mean scores of the CB component were entered in a one-way rm-ANOVA, with time (T1, T2, T3, T4) as a within-subjects factor. The results showed a significant main effect of time ($F(3, 117) = 4.083$, $p < 0.013$). Post-hoc tests using Bonferroni correction indicated that the mean CB score at time T1 (M = 6.80, SD = 4.334), collected at the initial orientation session, was larger than the mean score at both time T2 (M = 5.05, SD = 3.721) and T3 (M = 5.22, SD = 3.899). These results are shown in Fig 1 (b).

Similarly, Fig 1 (c) shows that there was a significant main effect of time on the FI component ($F(3, 117) = 2.730$, $p < 0.047$), with post-hoc tests using Bonferroni correction indicating that the mean FI score at time T1 (M = 7.67, SD = 4.281) was larger than the mean score at time T2 (M = 5.90, SD = 3.875).

There was also a significant main effect of time on the mean scores for the TA component ($F(3, 117) = 4.954$, $p < 0.003$). Post-hoc tests using Bonferroni correction indicated that the mean TA score at time T1 (M = 8.65, SD = 4.554) was larger than the mean score at both time T2 (M = 6.68, SD = 4.129) and T3 (M = 6.43, SD = 3.974). These results are shown in Fig 1 (d).

Anger-Hostility (AH) and Depression-Dejection (DD) scores across time

There was no significant difference between the mean scores across time on either the AH component ($F(3, 117) = 2.505$, $p = 0.075$) or the DD component ($F(3, 117) = 1.128$, $p = 0.302$).

Satisfaction With Life Scale (SWLS) scores across time

Mean SWLS scores were entered in a one-way rm-ANOVA, with time (T1, T2, T3, T4) as a within-subjects factor. The results showed a significant main effect of time ($F(3, 117) = 12.201$, $p < 0.001$). Post-hoc tests revealed that the mean SWLS at T3 (M = 21.70, SD =

6.313) was greater than the mean score at both T1 (M = 18.60, SD = 6.997) and T2 (M = 19.25, SD = 6.392). Also, the mean score at T4 (M = 22.17, SD = 7.243) was greater than the scores at both T1 and T2. These results are shown pictorially in Fig 2.

Fig 2. SWLS across time at T1, T2, T3 and T4 (see caption for Fig. 1 for details). The asterisk (*) indicates statistically significant pairwise differences ($p < 0.05$ level), corrected for multiple comparisons using the Bonferroni method.

Gratitude Questionnaire (GQ6) scores across time

A one-way rm-ANOVA, with time (T1, T2, T3, T4) as a within-subjects factor, revealed a significant main effect for time on the mean scores on the Gratitude Questionnaire (GQ6) ($F(3, 117) = 3.482$, $p < 0.018$). However, post-hoc tests using Bonferroni correction for multiple comparisons failed to detect pairwise differences between samples collected at different time points.

Discussion

We sought to examine changes in the affective state of Japanese university students participating in a short-term study-abroad (STSA) program of one week duration, in which fieldwork activities by the visiting students were undertaken with the help of local students (in either Cambodia or Malaysia). We first provided confirmatory evidence that the individuals taking part in the program were a representative sample of students, using the NEO-Five Factor Inventory to establish that there were no significant differences in major personality traits between study-abroad participants and a comparison group drawn from across the university sector. We next employed POMS to assess the mood states of the study-abroad group, as defined by the categories Anger-Hostility, Confusion-Bewilderment, Depression-Dejection, Fatigue-Inertia, Tension-Anxiety and Vigor-Activity. Our results show that Vigor-Activity increased significantly during their stay overseas (T3), when compared with the levels

observed before their departure (T2) (see Fig 1(a)), and generally remained high throughout the following weeks (T4).

Mood states, as measured by scales such as the POMS, have been shown to be enhanced using a variety of different manipulations, including meditation [34-35], sitting isometric yoga [36], massage therapy [37], exposure to nature [38] and physical activities [39]. Here, we attribute the observed enhancements in the scores of Vigor-Activity to participation in the study-abroad program. In addition, using the Satisfaction With Life Scale (SWLS), we showed that participation in STSA programs, even when limited to one week duration, effected a significant improvement in a core component of subjective well-being that was sustained for at least a few weeks after the students returned from overseas (see Fig 2). However, contrary to our expectations, no differences in GQ-6 scores were detected.

We conclude that STSA programs limited in duration to just one week are capable of positively impacting the affective state of students. This is important because short-term programs provide a more accessible alternative to long-term programs in terms of both time and money, and because they are likely to be a realistic entry point for students who otherwise would not consider the possibility of obtaining overseas experience during their years in college. Continued validation of STSA programs using scales such as the BEVI (Beliefs, Events, Values Inventory), IDI (Intercultural Development Inventory) and GPI (Global Perspectives Inventory) (reviewed in [40]) will enable educators to construct more accurate expectations of the long-term benefits of participating in such programs. To gauge the relative value of the affective state change in students afforded by STSA programs, it may also be advantageous in future studies to monitor university students over an extended time-period (e.g., one year) to assess the level of variation in their affective state with other significant events in the college calendar (e.g., exam and holiday periods).

Declaration of conflicting interests

The authors declare no potential conflicts of interest with respect to the research, authorship and/or publication of this article.

References

1. Paige, R. M., & Vande Berg, M. Why students are and are not learning abroad: A review of recent research. In M. Vande Berg, M. R. Paige, & K. Hemming Lou (Eds.), Student learning abroad: What our students are learning, what they're not, and what we can do about it, 29-58. Sterling, VA: Stylus Publishing. 2012.
2. OECD. Indicator C4: Who studies abroad and where?, in Education at a Glance 2014: OECD Indicators, OECD Publishing, Paris. http://dx.doi.org/10.1787/888933118656
3. Horie, M. Japan. In De Wit, H., Junter, F., Howard, L., & Egron-Polak, E. (Eds.), Internationalisation of Higher Education. 2015.
4. Lewis, T. M., and Niesenbaum, R. A. Extending the Stay: Using Community-Based Research and Service Learning to Enhance Short-Term Study Abroad. Journal of Studies in International Education. 2005; 9(3).
5. Anderson, P. H., Lawton, L., Rexeisen, R. J., & Hubbard, A. C. Short-term study abroad and intercultural sensitivity: A pilot study. International Journal of Intercultural Relations. 2006; 30. https://doi.org/10.1016/j.ijintrel.2005.10.004
6. Dwyer, M. M. More is better: The impact of study abroad program duration. Frontiers: The Interdisciplinary Journal of Study Abroad. 2004;10.
7. Chieffo, L., & Griffiths, L. What's a month worth? Student perceptions of what they learned abroad. International Educator. 2003; 12(4).
8. Yokota, M., Ashizawa, S., & Ota, H., et.al. Gu ro o ba ru ji n za i i ku se i to ryu u ga ku no tyo u ki te ki na i n pa ku to ni ka n su ru cho u sa ho u ko ku sho (in Japanese, A investigation report on global human resource development and longtitutional impact of studying abroad) 2016. Retrieved from http://recsie.or.jp/wp-content/uploads/2016/04/Survey-on-study-abroad-impact_final20170529.pdf
9. Chieffo, L., & Griffiths, L. Large-scale assessment of student attitudes after a short-term study abroad program. Frontiers: The Interdisciplinary Journal of Study Abroad. 2004;10.

10. Kurt, M. R., Olitsky, N. H., & Geis, P. Assessing global awareness over short-term study abroad sequence: A factor analysis. Frontiers: The Interdisciplinary Journal of Study Abroad. 2013;23.
11. Nguyen, A. D., Jefferies, J., & Rojas, B. Short term, big impact? Changes in self-efficacy and cultural intelligence, and the adjustment of multicultural and monocultural students abroad. International Journal of Intercultural Relations. 2018; 66. https://doi.org/10.1016/j.ijintrel.2018.08.001
12. McCrostie, J. More Japanese may be studying abroad, but not for long. The Japan Times of Aug 9, 2017, available at https://www.japantimes.co.jp/community/2017/08/09/issues/japanese-may-studying-abroad-not-long/#.Xi5brlP7St8 (Last retrieved on Feb 19, 2020)
13. Twombly, S., Salisbury, M. H., Tumaunt, S. D., & Klute, P., Study abroad in a new global century: Renewing the promise, refining the purpose. ASHE Higher Education Report. 2012;38(4).
14. Savicki, V. & Brewer, E. (Eds.) Assessing study abroad: Theory, tools, and practice, Sterling, VA: Stylus Publishing. 2015.
15. Costa. P .T., Jr., & McCrae. R. R. Revised NEO Personality Inventory (NEO-PI-R) and NEO Five-Factor Inventory (NEO-FFI) professional manual. Odessa, FL: Psychological Assessment Resources, Inc. 1992.
16. Chadyuk, O. NEO five-factor inventory and its applications in industrial. Organizational Psychology. 2015. https://doi.org/10.13140/RG.2.1.2126.8648
17. Spence, R., Owens, M., & Goodyer, I. Item response theory and validity of the NEO-FFI in adolescents. Personality and Individual Differences. 2012;53. https://doi.org/10.1016/j.paid.2012.06.002
18. De Fruyt, F., De Bolle, M., McCrae, R. R., Terracciano, A., Costa, P. T., Jr., & Collaborators of the Adolescent Personality Profiles of Cultures, P. Assessing the universal structure of personality in early adolescence: The NEO-PI-R and NEO-PI-3 in 24 cultures. Assessment. 2009;16. https://doi.org/10.1177/1073191109333760

19. McCrae, R. R., Costa, P. T., Jr., & Martin, T. A. The NEO-PI-3: A more readable revised NEO Personality Inventory. Journal of Personality Assessment. 2005;84. https://doi.org/10.1207/s15327752jpa8403_05
20. McCrae, R. R., Costa, P. T., Jr., Ostendorf, F., Angleitner, A., Hrebickova, M., Avia, M. D., Sanz, J., Sanchez-Bernardos, M. L., Kusdil, M. E., Woodfield, R., Saunders, P. R., & Smith, P. B. Nature over nurture: Temperament, personality, and life span development. Journal of Personality and Social Psychology. 2000;78.
21. Heuchert, J. P., & McNair, D. M. Profile of Mood States, 2nd Edition: POMS 2. North Tonawanda, NY: Multi-Health Systems Inc. 2012.
22. Bostock, S., Hamer, M., Wawrzyniak, A. J., Mitchell, E. S., & Steptoe, A. Positive emotional style and subjective, cardiovascular and cortisol responses to acute laboratory stress. Psychoneuroendocrinology. 2011;36. https://doi.org/10.1016/j.psyneuen.2011.02.009
23. Yoshioka, M., Ayabe, M., Yahiro, T., Higuchi, H., Higaki, Y., St-Amand, J., Miyazaki, H., Yoshitake, Y., Shindo, M., & Tanaka, H. Long-period accelerometer monitoring shows the role of physical activity in overweight and obesity. International Journal of Obesity. 2005;29. https://doi.org/10.1038/sj.ijo.0802891
24. Yokoyama, K., Araki, S., Kawakami, N., & Tkakeshita, T. Production of the Japanese edition of profile of mood states (POMS): assessment of reliability and validity. Nihon Koshu Eisei Zasshi. 1990;37.
25. Brown, H. E. Sojourner adjustment among undergraduate students: Relationships with locus of control and coping strategies. Michigan: UMI Dissertation Services. 1989.
26. Diener, E., Emmons, R. A., Larsen, R. J., & Griffin, S. The Satisfaction With Life Scale. Journal of Personality Assessment. 1985;49(1).
27. Diener, E. Subjective Well-Being. Psychological Bulletin. 1984;95(3).
28. Pavot, W. & Diener, E. Review of the satisfaction with life scale. Psychological Assessment 1993;5(2).
29. Whisman, M. A. & Judd, C. M. A cross-national analysis of measurement invariance of the Satisfaction With Life Scale. Psychological Assessment. 2016; 28(2).

30. McCullough, M. E., Emmons, R. A., & Tsang, J.-A. The grateful disposition: A conceptual and empirical topography. Journal of Personality and Social Psychology. 2002; 82(1).
31. Wood, A. M., Joseph, S., Maltby, J. Gratitude uniquely predicts satisfaction with life: Incremental validity above the domains and facets of the five factor model. Personality and Individual Differences. 2008;45(1).
32. Witvliet, C. v., Richie, F. J., Root Luna, L. M., VanTongeren, D. R. Gratitude predicts hope and happiness: A two-study assessment of traits and states. Journal of Positive Psychology. 2019;14(3).
33. Waters, L. Predicting job satisfaction: Contributions of individual gratitude and institutionalized gratitude. Psychology. 2012;3(12A).
34. Edwards, M. K. & Loprinzi, P. D. Experimental effects of brief, single bouts of walking and meditation on mood profile in young adults. Health Promotion Perspectives. 2018;8(3).
35. Travis, F., Valosek, L., Konrad, A., Link, J., Salerno, J., Scheller, R., & Nidich, S. Effect of meditation on psychological distress and brain functioning: A randomized controlled study. Brain and Cognition. 2018;125.
36. Oka, T., Tanahashi, T., Sudo, N., Lkhagvasuren, B. & Yamada, Y. Changes in fatigue, autonomic functions, and blood biomarkers due to sitting isometric yoga in patients with chronic fatigue sydnrome. BioPsychoSocial Medicine. 2018;12(3).
37. Donoyama, N., Satoh, T., Hamano, T., Ohkoshi, N. & Onuki, M. Effects of Anma therapy (Japanese massage) on health-related quality of life in gynecologic cancer survivors: A randomized controlled trial. PLOS One. 2018;13(5):e0196638.
38. Song, C., Ikei, H., Park, B.-J., Lee, J., Kagawa, T., & Miyazaki, Y. Psychological Benefits of Walking through Forest Areas. International Journal of Environmental Research and Public Health. 2018;15(12).
39. Crush, E. A., Frith, E., Loprinzi, P. D. Experimental effects of acute exercise duration and exercise recovery on mood state. Journal of Affective Disorders. 2018;229.
40. Roy, P., Wandschneider, E., & Steglitz, I.. Assessing education abroad outcomes: A review of the BEVI, IDI, and GPI. White Paper. 2014.

Click here to access/download;Figure;Fig1.tiff

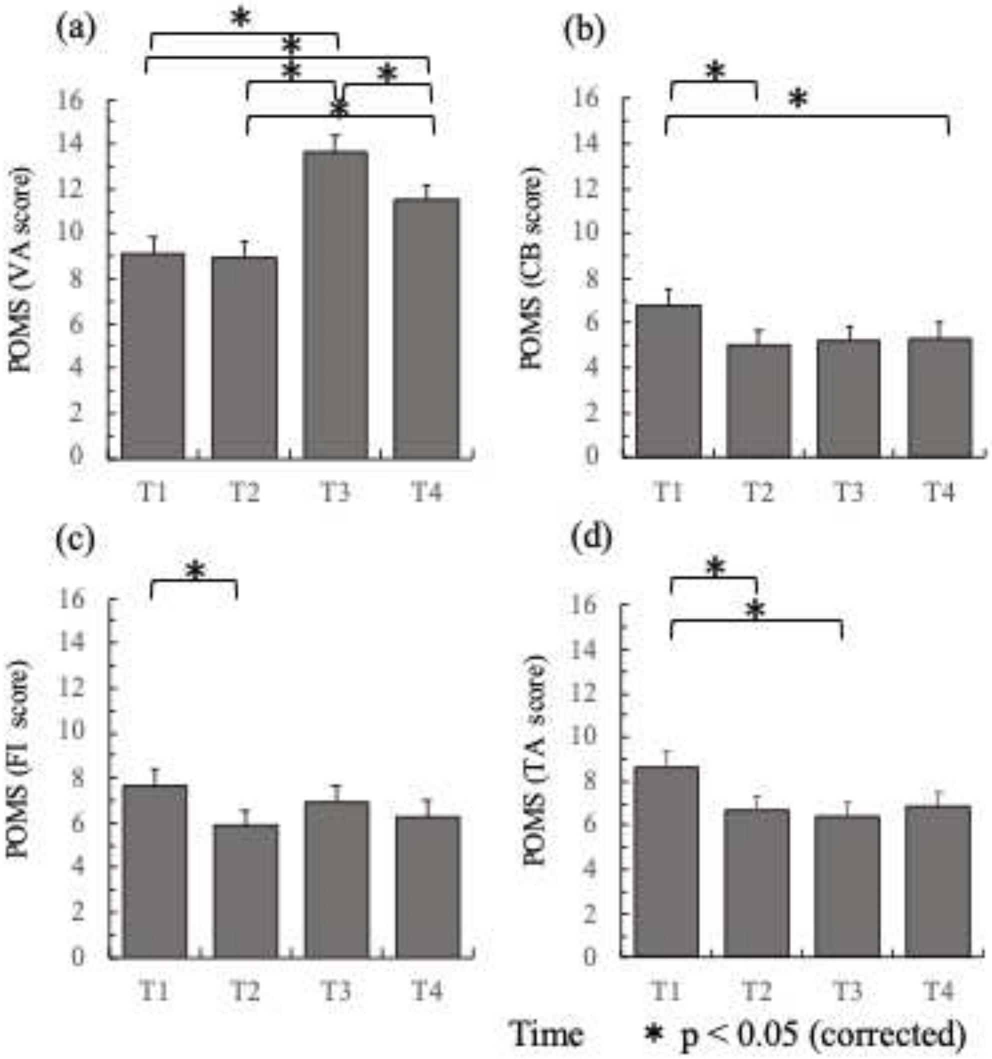

Click here to access/download;Figure;Fig2.tiff

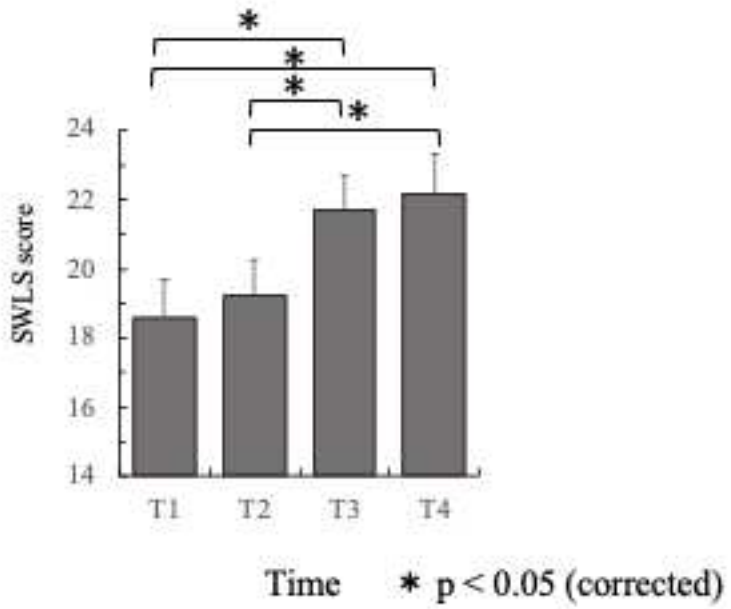

Supplement: Attachment — Submitted filename: PONE-D-21-12484_reviewer (1).pdf [file pone.0261762.s002.pdf]
