# Supplementary material for: Assessing changes in mood state in university students following short-term study abroad
Source: PLoS One. 2021 Dec 23;16(12):e0261762. doi: 10.1371/journal.pone.0261762 (PMC8699600; doi:10.1371/journal.pone.0261762)
Supplement: Attachment — Submitted filename: Reply_to_Reviewers_Comments_PONE-D-21-12484R1_24 Nov 2021.docx [file pone.0261762.s004.docx]

November 24, 2021

Dear Prof. Berta Schnettler,

Academic Editor

PLOS ONE

Re: PONE-D-21-12484R1

Thank you for giving us the opportunity to submit a revision of our manuscript. We greatly appreciate the time and effort that you and the reviewers have made to improve our submission, and are grateful for receiving such supportive and informative feedback.

In the revised manuscript, we have addressed your suggestions and the reviewers’ comments. In particular, following the comments of Reviewer 1, we now cite the empirical evidence that does not support Lysgaard’s U-curve hypotheses in the Introduction. Additionally, as requested, reference to the U-curve has been removed from the Discussion, while more emphasis has been placed on the issue of re-entry shock. We thank Reviewer 1 for this insightful direction.

A point-by-point response is given below. We include a clean copy of the revised manuscript, plus a copy highlighting all the changes that have been completed. If there is further need for clarifications, please do not hesitate to contact us.

We hope that our paper will now be acceptable for publication in PLOS ONE, and look forward to hearing from you.

With very best wishes,

T. Yamanaka, PhD, on behalf of the authors.

---***---***---***---***---***---***---***---***---

COMMENTS FROM/REPLIES TO REVIEWER #1

---***---***---***---***---***---***---***---***---

Reviewer #1:

Although I appreciate the authors’ careful attention to each aspect of the reviewers’ comments, I remain concerned about the rationale for studying mood in STSA. In their revision, the authors have added a discussion of Lysgaard’s U-curve hypotheses (in the Introduction and the Discussion sections) as central to their rationale. Yet, empirical research has not supported the U-curve hypothesis (see, for example, Chien, 2016; Gray & Savicki, 2015). As I read the revision, I found myself still wondering about the possible significance and implications of the authors’ findings on STSA participants’ elevated mood. The statement in the Discussion indicating that “This is important because short-term programs are a more accessible alternative to long term…” still didn’t answer my question. Then, in the last paragraph of the manuscript, the authors make a fascinating comment – “[these findings] suggest that the SA participants did not suffer a reverse culture shock in the aftermath of the STSA, in contrast with what has been reported about long-term SA program participants.” It seems to me that this latter point is the more significant contribution of this study. If, in fact, STSA programs are characterized by less severe re-entry shock than their longer counterparts, this information could contribute to our understanding of why re-entry shock occurs and may shape the content of pre-departure and in-country orientation for STSA participants. To my knowledge, re-entry shock in STSA is a topic that has received little attention in terms of empirical investigations. I suggest one more revision in which any reference to the U-curve is removed and emphasis is placed on the issue of re-entry shock (as indicated by the assessment of mood). I believe that doing so could significantly strengthen this contribution to the study abroad literature.

Chien, Y.-Y. G. (2016). After six decades: Applying the U-curve hypothesis to the adjustment of international postgraduate students. Journal of Research in International Education, 15(1), 32-51. <https://doi.org/10.1177%2F1475240916639398>

Gray, K. M., & Savicki, V. (2015). Study abroad reentry: Behavior, affect, and cultural distance. Frontiers: The Interdisciplinary Journal of Study Abroad, 26, 264-278. <https://doi.org/10.36366/frontiers.v26i1.370>

We thank the Reviewer for the useful suggestions made for improving our manuscript. As requested, we have now added arguments and supporting material about reverse culture shock after STSA, both in the Introduction and Discussion. Additionally, we now cite the empirical evidence that does not support Lysgaard’s U-curve hypotheses in the Introduction, and reference to the U-curve has been removed from the Discussion. In addition to citing Chien (2016) and Gray et al (2015), three other references were added to the manuscript. References added are as follows:

[22] Gullahorn JT, Gullahorn JE. An Extension of the U-Curve Hypothesis. Journal of Social Issues. 1963;19(3):33-47.
→ Cited in the 7^th^ paragraph of "Introduction".

[23] Ward C, Okura Y, Kennedy A, Kojima T. The U-Curve on trial: A longitudinal study of psychological and sociocultural adjustment during the Cross-Cultural transition. Cross-cultural transition. 1998.
→ Cited in the 7^th^ paragraph of "Introduction".

[24] Chien Y-YG. After six decades: Applying the U-curve hypothesis to the adjustment of international postgraduate students. Journal of Research in International Education. 2016;15(1):32-51.
→ Cited in the 7^th^ paragraph of "Introduction".

[25] Martin JN, Harrell T. Intercultural reentry of students and professionals: theory and practice. In Handbook of Intercultural Training. Publications Inc. 2004. p. 309-336
→ Cited in the 7^th^ paragraph of "Introduction".

[26] Gray KM, Savicki V. Study Abroad Reentry: Behavior, Affect, and Cultural Distance. Frontiers: The Interdisciplinary Journal of Study Abroad. 2015 ;26(1).
→ Cited in the 7^th^ paragraph of "Introduction".
→ Cited in the 9^th^ paragraph of "Discussion".

We hope all your suggestions and concerns were properly addressed in the revised manuscript. Thank you very much for your time and effort in improving our paper.

---***---***---***---***---***---***---***---***---

COMMENTS FROM/REPLIES TO REVIEWER #2

---***---***---***---***---***---***---***---***---

Reviewer #2: The authors have addressed most of my concerns and the revised paper is much clearer. Although the author refused to provide descriptive statistics, I am fine with that as long as the editor and the journal don't require that. The paper will contribute to the field of SA and I recommend it for publication.

Thank you for recommending our manuscript for publication in PLOS ONE. Note that all raw data is provided, and in the main text we have reported rigorous and appropriate statistical analyses of all results, which we believe are in line with Journal policy.

--***---***---***---***---***---***---***---***---

MINOR CORRECTIONS

--***---***---***---***---***---***---***---***---

A minor correction was made to the name of ethics committee. Plus we now make it explicit that ‘written’ informed consent was obtained prior to participation in the study.
